# Disentangling Cerebellar and Parietal Contributions to Gait and Body Schema: A Repetitive Transcranial Magnetic Stimulation Study

**DOI:** 10.1007/s12311-024-01678-x

**Published:** 2024-03-05

**Authors:** Margherita Bertuccelli, Patrizia Bisiacchi, Alessandra Del Felice

**Affiliations:** 1 Department of Neuroscience, Section of Neurology, University of Padova, Padua, Italy; 2 Padova Neuroscience Center, University of Padova, Padua, Italy; 3 Department of General Psychology, University of Padova, Padua, Italy

**Keywords:** Non-invasive brain stimulation (NIBS), Inertial measurement unit, Sensorimotor integration, Ataxia, Posterior parietal lobe

## Abstract

The overlap between motor and cognitive signs resulting from posterior parietal cortex (PPC) and cerebellar lesions can mask their relative contribution in the sensorimotor integration process. This study aimed to identify distinguishing motor and cognitive features to disentangle PPC and cerebellar involvement in two sensorimotor-related functions: gait and body schema representation. Thirty healthy volunteers were enrolled and randomly assigned to PPC or cerebellar stimulation. Sham stimulation and 1 Hz-repetitive-Transcranial-Magnetic-Stimulation were delivered over P3 or cerebellum before a balance and a walking distance estimation task. Each trial was repeated with eyes open (EO) and closed (EC). Eight inertial measurement units recorded spatiotemporal and kinematic variables of gait. Instability increased in both groups after real stimulation: PPC inhibition resulted in increased instability in EC conditions, as evidenced by increased ellipse area and range of movement in medio-lateral and anterior–posterior (ROMap) directions. Cerebellar inhibition affected both EC (increased ROMap) and EO stability (greater displacement of the center of mass). Inhibitory stimulation (EC vs. EO) affected also gait spatiotemporal variability, with a high variability of ankle and knee angles plus different patterns in the two groups (cerebellar vs parietal). Lastly, PPC group overestimates distances after real stimulation (EC condition) compared to the cerebellar group. Stability, gait variability, and distance estimation parameters may be useful clinical parameters to disentangle cerebellar and PPC sensorimotor integration deficits. Clinical differential diagnosis efficiency can benefit from this methodological approach.

**Supplementary Information:**

The online version contains supplementary material available at 10.1007/s12311-024-01678-x.

## Introduction

Sensorimotor integration is the process whereby different sources of sensory inputs are integrated by the central nervous system to guide motor program execution [1]. Proprioceptive and visual signals integration is critical for efficient locomotion: vision is primarily used to explore the environment, identify obstacles, and their locations relative to the body, while proprioception provides constantly updated information on body segment positions [2]. Two brain areas are mainly responsible for integrating multisensory information pertaining to gait: the posterior parietal cortex (PPC) and the cerebellum [3]. PPC receives inputs from visual cortices shaping the dorsal stream (“vision-for-action” pathway), which is involved in the real-time control of actions [4]. PPC integration of visual signals with proprioceptive ones allows transforming spatial location, orientation, and motion of objects into the coordinate frames of the motor effectors [5]. This evidence serves as the foundation for the PPC's involvement in the creation of the body schema representation: an unconscious and dynamic representation of the body position in space and the configuration of its parts with respect to one another and the outside environment. [6, 7].

Similarly, the cerebellum plays a role in integrating multisensory cortical and subcortical inputs [8]. The integration of signals from different peripheral receptors and motor cortices [9] is thought to be part of the feedback and feedforward error detection processes involved in online motor adjustments [10]. Involvement of the cerebellum in encoding limb spatial position has also been proposed [10], which points to its potential role in body schema determination.

Besides their functional similarities in motor control, lesions in both these brain structures have been associated with cognitive deficits in visuomotor integration, spatial cognition, working memory, and expressive language [11–13].

Current knowledge on PPC and cerebellar contributions to sensorimotor integration focuses mainly on reach-to-grasp movements [14] and often relies on studies conducted independently on people with parietal or cerebellar lesions. The overlap between motor and cognitive deficits resulting from PPC or cerebellar lesions can mask their relative contribution to sensorimotor integration processes. This often prevents a straightforward localizing diagnosis [15]. Thus, disentangling their relative contribution has both a clinical and a theoretical rationale.

This study aims to define distinguishing motor and body schema-related parameters to disentangle PPC and cerebellar involvement in two sensorimotor-related functions: gait and body schema. Specific aims were: 1) identify stability, spatiotemporal, and kinematic parameters associated with either PPC or cerebellar functional inhibition; 2) assess the potential of a body schema-related task to discriminate PPC and cerebellar contributions to sensorimotor integration.

## Material and Methods

This study was a two-by-two factorial design, with a between group factor (i.e., stimulated brain region: PPC and cerebellum) and a within group factor (i.e., stimulation type: sham and real stimulation). The study was conducted following the guidelines of the Declaration of Helsinki and was approved by the ethical committee of the Department of General Psychology, University of Padua (protocol N.4562).

### Participants

**Fig. 1:**
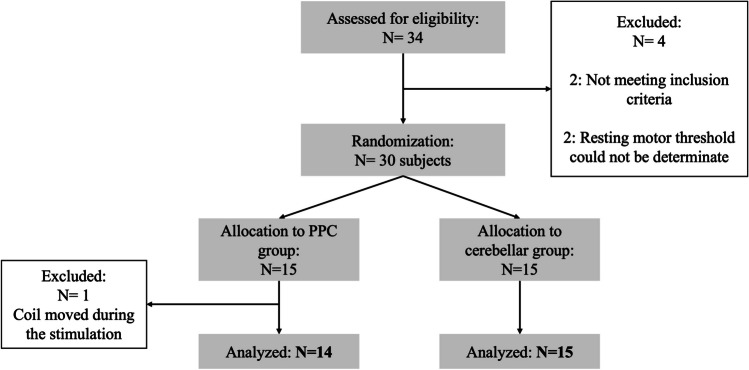
Flowchart of subjects’ random allocation to the stimulation condition. Schematic representation of participants’ enrollment process and random allocation to either PPC (i.e., 15 participants) or cerebellar group (i.e., 15 participants)

Thirty participants (21 females; mean ± SD age: 23.4 ± 2.9; range: [19-31]) recruited among the Psychology Department students of Padova University between March and June 2022, took voluntarily part in the study and provided written informed consent. All were right-handed and had normal or correct to-normal vision. Inclusion criteria comprised no neurological, psychiatric, or medical condition contraindicating transcranial magnetic stimulation (TMS), [16]. Exclusion criteria consisted of diagnosed gait alterations or movement abnormalities and orthopedic pathologies. After TMS eligibility assessment, 15 participants were randomly allocated to the PPC and 15 to the cerebellar stimulation group (see Fig. 1). Randomization was ensured by assigning each subject a number reflecting the enrollment order: odd numbers were allocated to the PPC group and even numbers to the cerebellar one. Each subject received the sham and the real stimulations within a single-day session in a counterbalanced manner. Participants were blind to stimulation conditions.

### Repetitive Transcranial Magnetic Stimulation (rTMS)

rTMS was delivered using a Magstim-Rapid^2^ stimulator with a D70^2^ B.I. air-cooled figure-of-eight coil allowing long stimulation sessions. Left PPC and right cerebellum were chosen as targets: left PPC seems to play a general role in walking in the real-world and visuomotor adaptations [17, 18] while right cerebellum (i.e., VIII-A lobule of the posterior cerebellum) is reachable by TMS and associated with motor functions [19]. Each subject underwent two sessions of stimulation within the same day:

1. The rTMS [1500 pulses, 1 Hz frequency at 90% intensity of the individual resting motor threshold (rMT)] was delivered over either the PPC or cerebellum. Low-frequency rTMS (≤ 1 Hz) has been proven to have inhibitory effects, with an after-effect length proportional to the length of the stimulation period [20–23].
2. A sham coil delivered a control stimulation over the same areas and for the same time.

The order of stimulation sessions was counterbalanced across participants. To control for possible carry-over effects, 40 min interspersed the two sessions (i.e., 20 min longer than the estimated after-effects of the stimulation [20–22]).

The rMTs were assessed via motor-evoked potentials (MEPs) by delivering single-pulse TMS over the primary motor cortex (M1). The coil was placed tangentially to the scalp with the handle pointing backwards and laterally at 45° away from the sagittal axis [24]. The elicited muscular activity was recorded over the right hand's first dorsal interosseus muscle (FDI). The minimum output intensity leading to 5 MEPs in 10 consecutive trials was selected as individual rMT. The rTMS stimulation intensity was eventually set at 90% of the rMT. The coil was positioned tangentially to the scalp over P3 to localize the left PPC, according to the international 10–20 EEG coordinate system [25]. To target the cerebellum, we positioned the coil 1 cm inferior and 3 cm lateral to the inion, following previous studies’ procedures [26, 27]. In this case, the coil was positioned tangentially to the scalp with the handle directed upwards: this was shown to be the optimal coil orientation to reach the cerebellum [19]. We did not opt for a double-cone coil to stimulate the cerebellum, as suggested in other works [26], as this was proved to induce invasive stimulation, often leading to pain and discomfort in neck muscles [28].

### Inertial Measurement Units (IMUs)

**Fig. 2:**
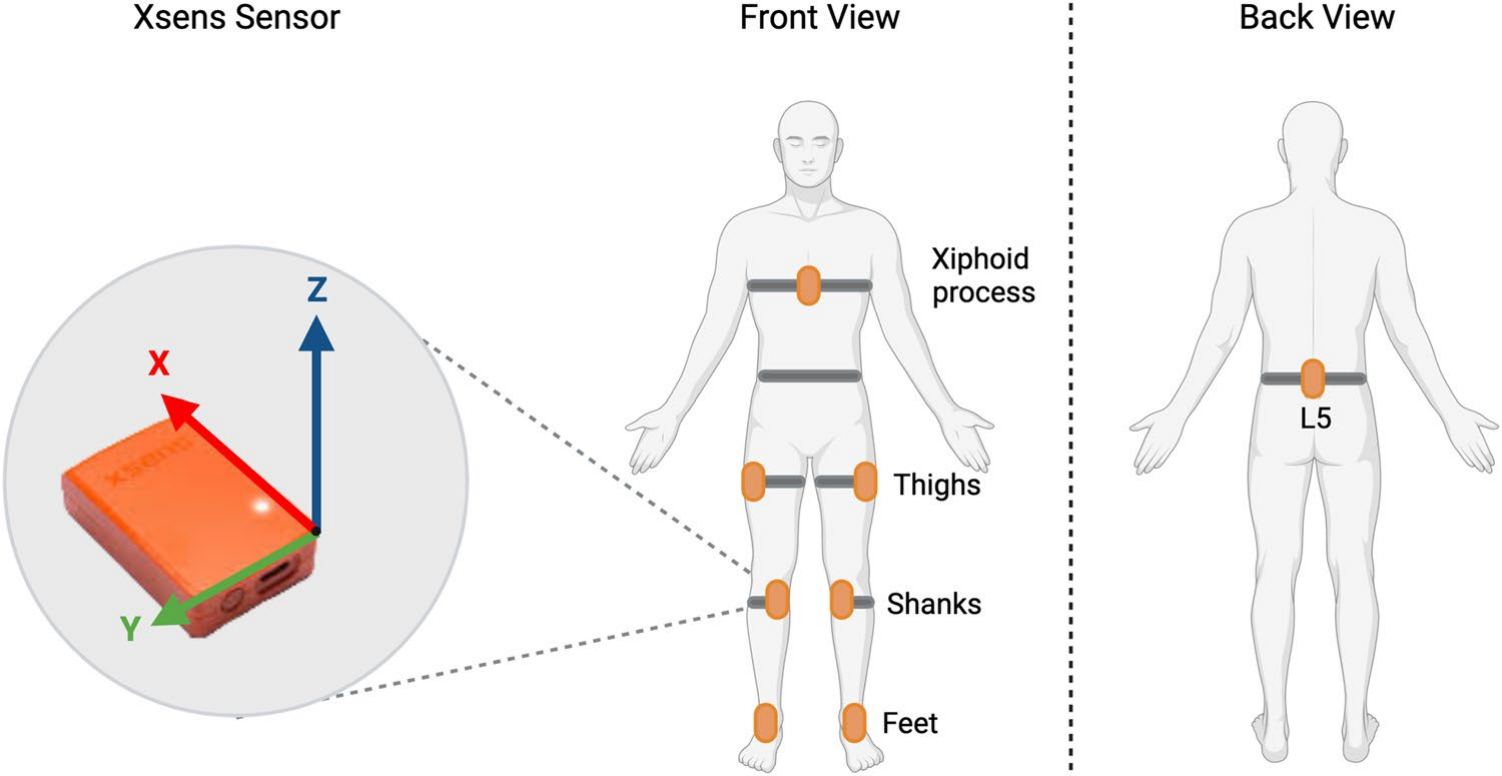
Xsens sensors positioning. Participant lower body plus sternum set-up, with 8 Xsens inertial measurement units (IMUs)

Participants were equipped with eight synchronized Xsens MTw IMUs (Xsens technologies, Enschede, Netherlands) secured with straps respectively on the sternum (xiphoid process), pelvis (vertebra L5), thighs (left and right trochanters), shanks (left and right proximal medial frontal aspect), and feet (see Fig. 2). The sensors provide filtered and strapped-down samples of acceleration, angular velocity and magnetic rate vectors, as well as the estimated quaternion of orientation [29]. Anthropometric parameters were gathered for every subject, including weight, sole shoe height, and foot length. IMUs sensors were calibrated following the recommended stand still and walking procedure allowing the software model to establish a relation between sensors and segment orientation [29, 30].

Postural stability was assessed during the execution of the Romberg’s Test through the following parameters: center of mass (COM) path length trajectory (PL); ellipse area containing 95% of the COM points (EA); COM range of movement (ROM) in anterior–posterior (ROMap) and medio-lateral directions (ROMml); root mean square (RMS) of the COM positions in anterior–posterior (RMSap) and mediolateral directions (RMSml). Gait spatiotemporal parameters of interest were the following: cadence (step/min), speed (m/sec), stance and swing times (sec), single and double support time (sec), stride and step time (sec), stride and step length (m), and step width (cm). To evaluate lower body kinematics, we considered hip, knee, and ankle joint angular displacement in the sagittal plane. Both spatiotemporal and kinematic parameters were extracted by analyzing gait during the distance estimation trials described in Section “Tasks”.

For each parameter mentioned above, we quantify within-subject variability. A widely used measure of variability is the coefficient of variation (CV), defined as the ratio of the standard deviation to the mean. Thus, the CV value is susceptible to outliers and assumes a normal population sample distribution [31]. On the contrary, the interquartile range (IQR), defined as the difference between the 75th and 25th percentiles of the data, does not require a normality assumption, and it is, therefore, more robust to the presence of outliers [31]. Considering this, we opted for IQR as a measure of data variability. All the parameters were normalized by individual height, weight, and feet length via a detrending normalization technique [32].

### Tasks

**Fig. 3:**
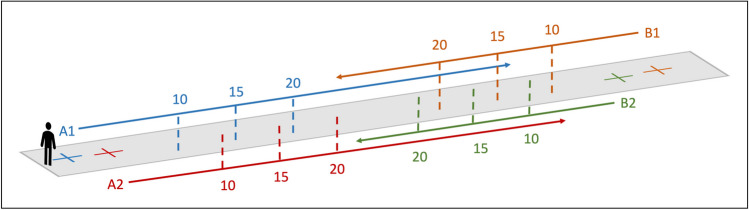
Distance estimation task. Schematic representation of the distance estimation task conditions. A1, A2, B1 and B2 represent the possible participants starting positions. The target could be presented at 10, 15 or 20-m distances from each starting position

Each of the following tasks was executed by donning the IMUs after the sham and the real stimulations in the following order:

1. Balance assessment: Romberg’s Test is a neurological assessment to evaluate postural instability and ataxia [33]. A re-adaptation of Romberg’s test was used to assess balance before and after the stimulation. Specifically, participants were asked to stand still for 10 s, keeping their feet together, with their arms alongside the body, keeping their eyes open and, during a second trial, closed. For both eyes open and closed trials, the COM path length trajectory, ellipse area containing 95% of the COM points, COM range of movement and root mean square positions in anterior–posterior and mediolateral directions were extracted as measures of stability.
2. Body schema assessment: a walking distance estimation task was used to assess possible body schema alterations. At the beginning of each trial, participants were asked to settle in one of four possible starting positions, keeping their eyes closed till the start of each trial. Once the experimenter instructed them to open their eyes, a target was presented for 3 s at three possible distances from the starting position (i.e., 10, 15, and 20 m). Varying target distances and starting positions prevented possible learning effects. Immediately after the target was removed, the participant was instructed to walk till the estimated target position was reached. (See Fig. 3). No feedback was provided about the performance at the end of each trial. Half of the trials were executed with the eyes open (12 trials) and half with the eyes closed (12 trials) after both stimulations. Once the participant reached the estimated target position, one experimenter measured the distance travelled from the starting position through a laser distance meter. Eyes conditions, distances, and starting conditions were randomized in RStudio software. The IMUs data were utilized to derive spatiotemporal and kinematic parameters associated with eyes open and eyes closed walking abilities for each walking distance estimation session.

### Data Analysis and Statistics

**Fig. 4:**
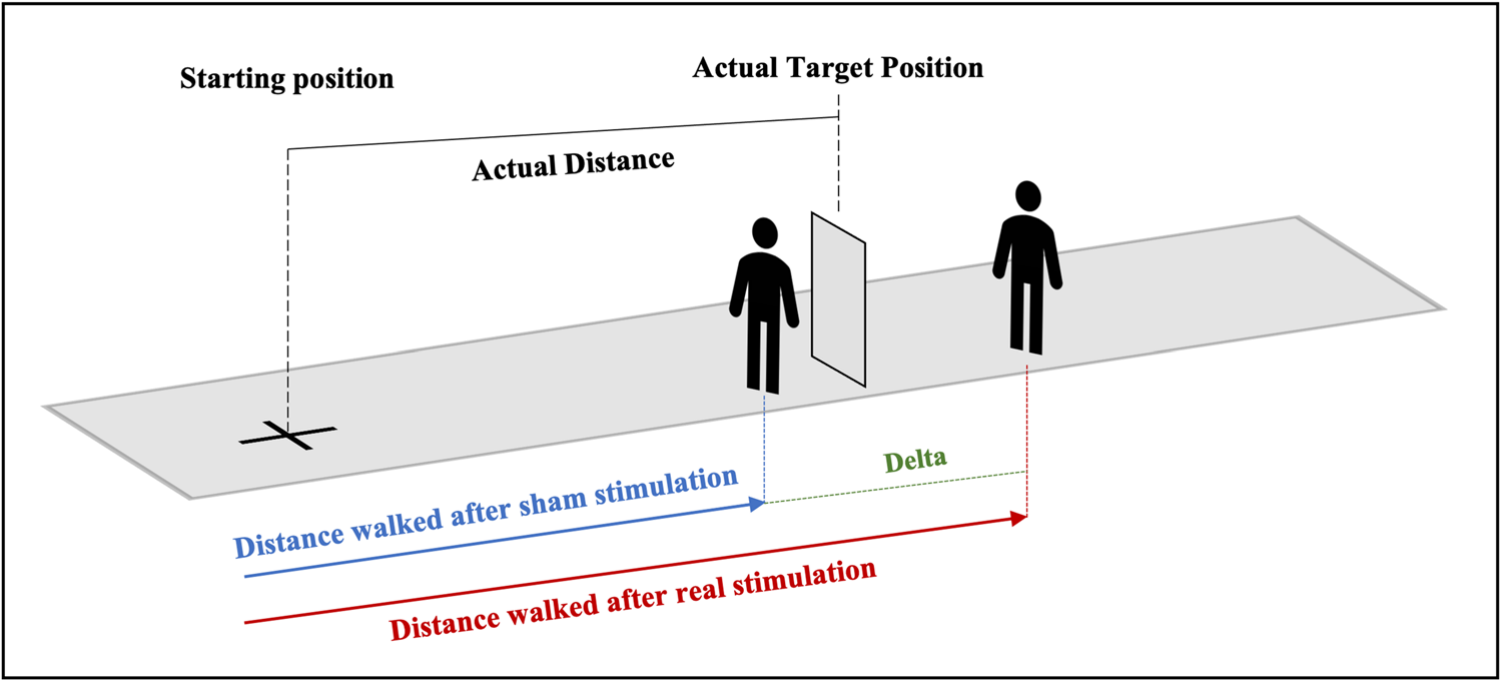
Schematic representation of delta values computation method. Delta values were calculated as the difference between the path travelled after real stimulation in meters (red line) and sham stimulation (blue line). Trials were clustered and analyzed separately by distance (10 m, 15 m, 20 m) and eye condition (open, closed)

1. Balance and gait: a custom post-processing algorithm was developed in MATLAB-R2020b. Xsens output files were processed to identify gait events [i.e., heel strike (HS) and toe-off (TO), [34]] for each trial. The HS and TO events detection were based on knee and ankle sagittal angle functions. The events were used to identify gait cycles for the right and left side. The first and last two meters of each trial were removed from the analysis to avoid confounding effects from starting and stopping at the edges of the walkway [35].
2. Distance estimation task: to control for inter-individual variability in the ability to estimate distances, we considered the performances in the sham trials as measures of the actual individual ability to estimate distances. Then, we computed the difference between the distance travelled after the real stimulation and the distance travelled after the sham stimulation grouping for distance and eye condition (i.e., EO: 10, 15, 20 m; EC:10, 15, 20 m). See Fig. 4 for a schematic representation. Delta values were used as indices of the stimulation effect on the ability to estimate distances, with delta = 0: no effect of stimulation; delta > 0: overestimation of distance; delta < 0: underestimation of distance.

The statistical analysis was performed using the RStudio software (RStudio Team, 2015, Version 1.2.5001). Statistical significance was set at *p*-value  < 0.05. Data distribution was tested with a Shapiro-Wilks normality test.

The primary outcome was the within-subject change in variability (i.e., IQR) of walking metrics and balance between real and sham stimulation. Indeed, greater gait variability has been linked to both PPC and cerebellar disorders [36, 37]. Secondary outcomes were between group differences in balance, walking and distance estimation abilities after real stimulation and within group EO *vs.* EC differences. Balance, walking parameters and distance estimation data were tested for possible differences between groups (PPC *vs.* cerebellar group) through an unpaired two-sided Wilcoxon rank sum test. Within-group differences due to stimulation effects (sham *vs* real) and eyes effects (EO *vs* EC) were tested with a paired two-sided Wilcoxon rank sum test to account for repeated measures within the same group. Considering the exploratory nature of the study, no corrections for multiple comparisons were performed in hypothesis testing.

## Results

Of the 30 initially enrolled participants, one of the PPC group was excluded from the analysis as the TMS coil moved from the target region during the real stimulation. See Table 1 for demographics related to the final analyzed sample.

**Table 1:** Demographic characteristics of the sample

Parameter	Units	PPC	Cerebellar
Age	*years*	24 ± 2,95	22 ± 2,79
Females	*%*	64%	73%
Body Mass	*kg*	64,14 ± 12,30	66,89 ± 12,30
Height	*m*	1,71 ± 0,10	1,71 ± 0,10
BMI ^a^	*kg/m*^*2*^	21,80 ± 2,24	22,59 ± 2,76
RMT ^b^	*%*	58%	59%
Nasion-Inion	*cm*	35,10 ± 2,36	35,28 ± 2,14
A1-A2 ^c^	*cm*	34,92 ± 1,90	35,92 ± 1,77

^a^*BMI* body mass index

^b^*RMT* resting motor threshold

^c^A1-A2: distance between earlobe electrodes

The Shapiro normality test revealed no normal distribution for balance, spatiotemporal and kinematic variables, while normal distribution was observed for delta values in the 10 m (*p*-value  = 0.07) and 20 m (*p*-value  = 0.56) conditions of the distance estimation task. No normal distribution emerged for deltas of the 15 m condition (*p*-value  = 0.04).

### Balance assessment: Romberg’s Test

**Fig. 5:**
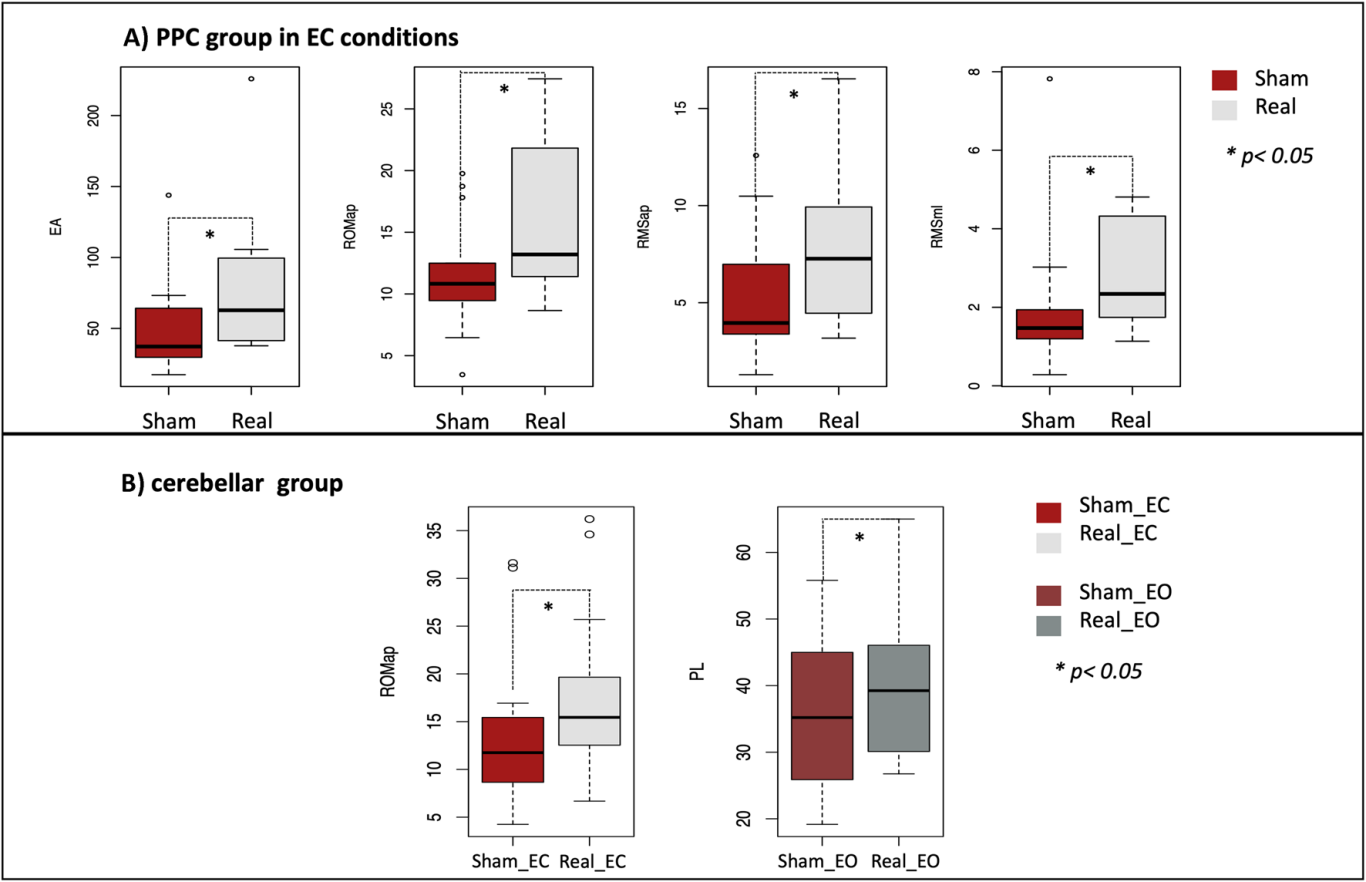
Romberg’s test boxplots. Significant differences in balance parameters of the PPC group (Panel **A**) and cerebellar group (Panel **B**) are represented. Panel **A**: PPC group show higher EA, ROMap, RMSap and RMSml in the real stimulation EC condition compared to the sham one (mean ± SD real EA: 78.22 ± 52.77 vs sham: 42.42 ± 22.97; real ROMap: 16.11 ± 6.66 vs sham: 12 ± 5.22; real RMSap: 8 ± 3.99 vs sham 5 ± 3.49; real RMSml: 3 ± 1. 29 vs sham: 2 ± 1,01). Panel **B**: Cerebellar PL resulted higher after real stimulation in the EO condition compared to the sham one (mean ± SD real PL: 40.46 ± 12.65 vs sham: 36.21 ± 12.32). Cerebellar ROMap increased after the real stimulation compared to sham in the EC condition (mean ± SD real ROMap: 18 ± 8.64, vs sham: 14 ± 7.65)

Figure 5 shows significant results of Romberg’s test. Real *vs.* sham stimulation in the EC condition within the PPC group showed higher EA (V = 56, *p*-value  = 0.04), ROMap (V = 89, *p*-value  = 0.02), RMSap (V = 85, *p*-value  = 0.04) and RMSml (V = 86, *p*-value  = 0.03). Similarly, the cerebellar group displayed higher ROMap after the real stimulation in the EC condition compared to the sham EC one (V = 98, *p*-value  = 0.03). The PL of the cerebellar group resulted longer after the real stimulation than the sham in the EO condition (V = 96, *p*-value  = 0.04). No significant between group differences emerged. All variables mean ± SD are reported in the supporting information, S1 Table.

### Gait: Spatiotemporal Parameters

PPC group significantly increased the variability (IQR) from sham EC condition to real EC condition in cadence (sham_mean ± SD: 2.69 ± 2.05 step/min, real: 4.39 ± 2.08 step/min, V = 12, *p*-value  = 0.01) and stride time (sham_mean ± SD: 0.05 ± 0.03 s, real: 0.07 ± 0.04 s, V = 12, *p*-value  = 0.01). Significant differences were observed between EO and EC conditions after real stimulation in the variability (IQR) of cadence (EO_mean ± SD: 2.36 ± 1.11 step/min, EC: 4.39 ± 2.08 step/min, V = 13, *p*-value  = 0.01), speed (EO_mean ± SD: 0.05 ± 0.02 step/min; EC: 0.11 ± 0.07 step/min, V = 16, *p*-value  = 0.02), stance time (EO_mean ± SD: 0.04 ± 0.04 s, EC: 0.06 ± 0.03 s, V = 14, *p*-value  = 0.02), and step time (EO_mean ± SD: 0.02 ± 0.01 s, EC: 0.04 ± 0.02 s, V = 10, *p*-value  < 0.01). No differences were observed between the EO and EC conditions after sham stimulation in the same variables. Swing time significantly increased after real stimulation in the EC condition (mean ± SD: 0.35 ± 0.02 s) compared to the EO one (mean ± SD: 0.04 ± 0.04 s, V = 12, *p*-value  < 0.01), while no differences were observed between EO and EC conditions after sham stimulation. The same pattern was observed for the step width, which increased after real stimulation in EC condition (mean ± SD: 12,29 ± 3,80 cm) compared to EO one (mean ± SD: 11,65 ± 4,20 cm, V = 19, *p*-value  = 0.03).

Cerebellar group significantly increased the variability (IQR) of the stride length after real stimulation in the EC condition compared to sham (sham_mean ± SD: 0.15 ± 0.06 m, real: 0.17 ± 0.06 m, V = 20 *p*-value  = 0.04). Cerebellar group’ speed variability (IQR) increased after real stimulation in the EC condition compared to the EO one (EO_mean ± SD: 0.07 ± 0.03 m/s, EC: 0.12 ± 0.08 m/s, V = 22, *p*-value  = 0.05) while no differences were observed between the EO and EC condition following the sham. In addition, step width increased after real stimulation in the EC condition (mean ± SD: 12,13 ± 3,89 cm), compared to the EO one (mean ± SD: 10,86 ± 3,43 cm, V = 8, *p*-value  < 0.01); no differences were observed between the equivalent sham conditions. No significant between-group differences were observed. All the spatiotemporal parameters (mean ± SD) of PPC and cerebellar groups are reported in Tables S2 of the supporting information.

### Gait: Kinematic Parameters

PPC group after real stimulation increased the variability (IQR) of the following kinematic parameters in the EC condition compared to the EO one: average knee angle (EO_mean ± SD: 1.98 ± 1.05 deg; EC: 2.21 ± 0.97 deg, V = 21, *p*-value  = 0.05), knee angle at foot elevation (EO_mean ± SD: 3.43 ± 1.31 deg; EC: 3.96 ± 1.22 deg, V = 11, *p*-value  < 0.01), ankle minimum angle (EO_mean ± SD: 4.07 ± 1.94 deg; EC: 4.97 ± 1.12 deg, V = 16, *p*-value  = 0.02), ankle angle at toe off event (EO_mean ± SD: 4.07 ± 1.94 deg; EC: 4.97 ± 1.12 deg, V = 16, *p*-value  = 0.02). No significant variations between sham EO *vs*. EC condition were observed for the same parameters.

Cerebellar group show increased variability (IQR) of the maximum ankle angle and of the knee angle at foot elevation after the real stimulation in the EC condition compared to the EO one, respectively: maximum ankle angle (EO_mean ± SD: 2.05 ± 0.58 deg; EC: 2.57 ± 0.84, V = 2, *p* < 0.01); knee angle at foot elevation (EO_mean ± SD: 3.77 ± 1.85 deg; EC: 5.24 ± 2.16,V = 16, p = 0.02). The average ankle angle variation increased from sham EC condition to real EC condition (sham_EC mean ± SD: 1.76 ± 0.97 deg; real_EC: 2.61 ± 1.72 1, V = 19, p = 0.02). No significant between-group differences were observed. All kinematic parameters (mean ± SD) of PPC and cerebellar group are reported in the supporting information S3 Table.

### Distance Estimation Task

**Fig. 6:**
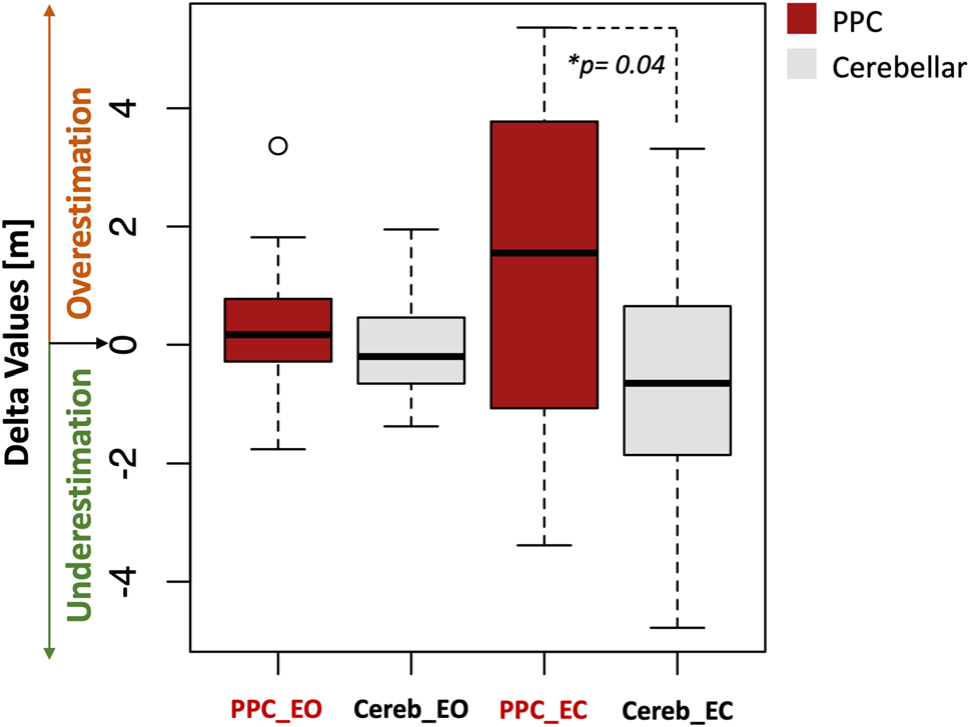
Distance estimation task boxplots. Boxplots represent the delta values of the 20 m trials. PPC group in the eyes closed trials (EC) tended to overestimate the target position [median error (1st-2nd quartile): 1.55 m (-0.84, 3.48)] significantly more than cerebellar group [median error (1st-2nd quartile): -0.64 m (-1.86, 0.65)]. No significant differences were observed between-groups in the trials performed with the eyes open [median error (1st-2nd quartile) PPC_EO: 0.047 m (-0.33, 0.62); median error cereb_EO: 0.20 m [-0.65, 0.46])

Significant difference emerged between PPC and cerebellar groups in the EC condition for the 20 m distance, with PPC group overestimating distances compared to cerebellar group (median error [range] of PPC in EC condition: 1.55 m [-0.84; 3.48]; Cerebellar in EC condition: -0.64 m [-1.86; 0.65], t = 2.09, *p*-value  = 0.04). Results are summarized in Fig. 6. For all conditions mean and median values see the supporting information, S4 Table.

## Discussion

**Fig. 7:**
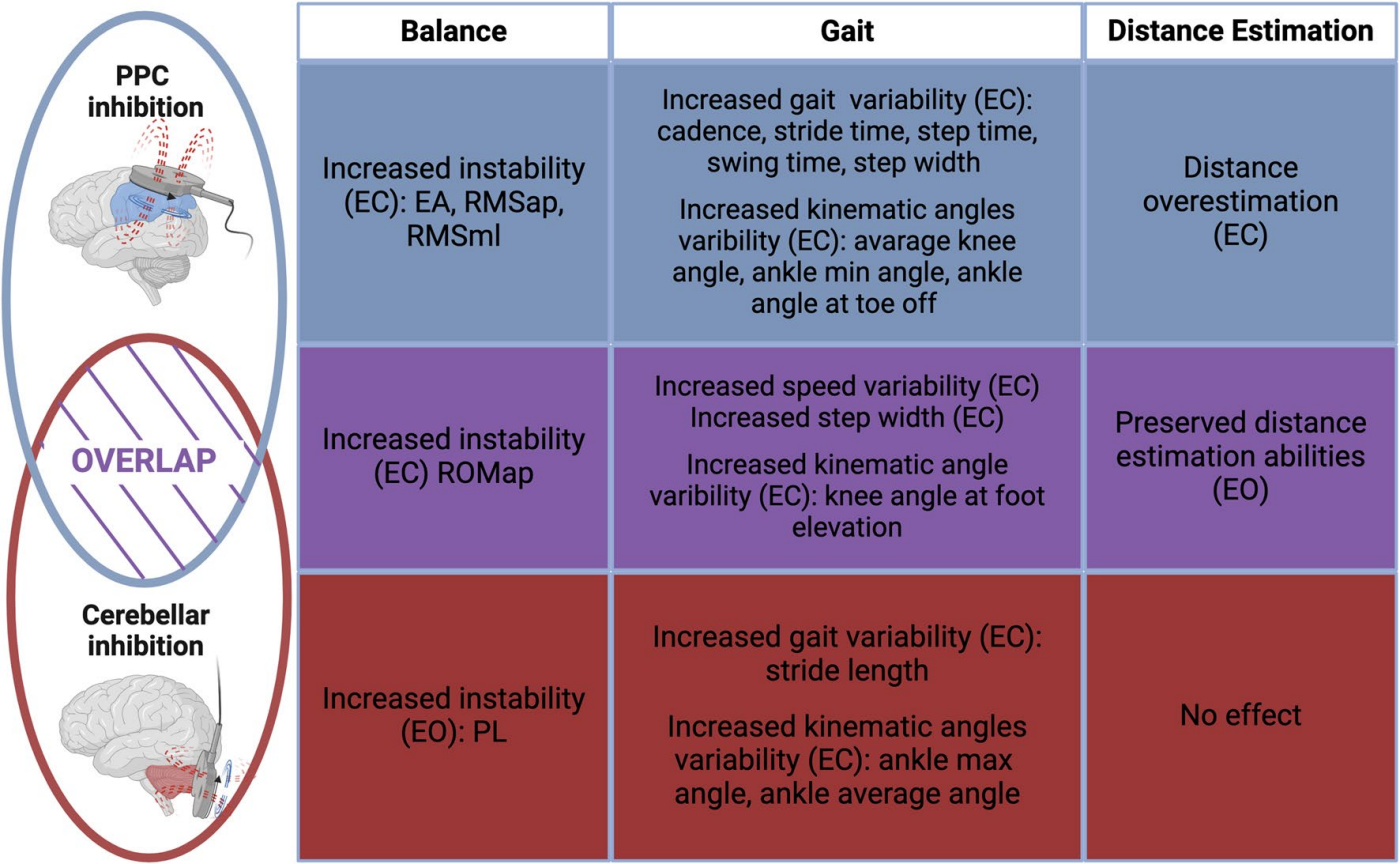
Summary of the results. Schematic representation of differences and overlaps of cerebellar and PPC contribution to balance control, gait, and distance estimation by walking task

This study addresses the functional contribution of PPC and cerebellum to gait and body schema. Specifically, we were interested in elucidating their roles employing an rTMS paradigm. Our data proved the potential of specific gait and stability parameters as well as the walking distance estimation task to differentiate PPC and cerebellar contribution to sensorimotor integration. The main findings on balance, spatiotemporal, kinematic and distance estimation parameters are summarized in Fig. 7. Particularly, our findings demonstrate that a walking distance estimation paradigm differentiates cerebellar *vs* PPC involvement and may be a transferrable, easy, and cost-effective task for clinical differential diagnosis.

We also report well-known signs related to cerebellar and PPC dysfunctions, as the irregular foot trajectories and increased variability of temporal and spatial gait parameters of cerebellar disorders and the correcting role of visual input to compensate for PPC related instability.

### PPC Inhibition Effects

PPC functional inhibition effects emerged when visual feedback was lacking. PPC group instability increased after real stimulation as measured by higher EA and increased ROM on the anterior–posterior and mediolateral axis in the EC condition. No differences between sham and real stimulations were observed with eyes open. These results align with previous studies showing effects resembling those of sensory ataxia (SA) on Romberg's test [33]. In SA, peripheral impairments of the somatosensory afferents lead to altered/interrupted sensory feedback used to track limb positions in space. This, in turn, leads to gait abnormalities (e.g., increased stepping width) and instability when visual compensation is hampered. PPC has a crucial role in integrating multisensory signals to provide coherent representations of our body in space (i.e., body schema) and to set proper motor outputs [38]. By functionally inhibiting the PPC, we likely interfered with the central process of sensory integration: the motor alterations we induced share clinical characteristics with SA. Particularly, while in SA the deficits arise for a lack of sensory feedback to the central nervous system, the functional inhibition of the PPC altered the central integration of these sensory feedback. This can explain why the PPC group balance was altered just in the absence of visual feedback. Secondly, cadence, speed, stance, and step time variabilities increased after the real stimulation, as well as the step width in the EC condition compared to the EO one. These gait characteristics can suggest an unsteady gait, which is akin to the pattern commonly seen in individuals with SA when visual information is lacking: general disturbances of sensory feedback and/or integration during walking (regardless of the sensory modality) have been proved to be tightly linked to increased spatiotemporal gait variability [8]. Altering PPC sensory integration processes leads to an altered perception of steps' timing and placing, resulting in higher temporal variability and a wider support base. Beside this, it is worth to point out that the walking was assessed within a distance estimation task rather than under typical walking circumstances. Consequently, a different explanation for the increased spatiotemporal gait variability following stimulation could be a change in the body's schema representation in general, which could result in inaccurate distance calculation and, consequently, in different step length and timing when walking to the target. Kinematic alterations in the PPC group affected mainly the ankle joint: we observed a wider ankle angle at heel strike, compatible with the so-called "slapping step" walking and higher variability of the knee angle at foot elevation, compatible with the "high stepping" pattern [39]. These results together with the increased variability in other ankle angles (i.e., minimum ankle angle, the angle at toe-off) and knee angles (i.e., average knee angle) may be again the result of a general alteration in the body schema leading to the inefficient ankle and knee placements while walking [40].

### Cerebellar Inhibition Effects

The cerebellar role in gait and balance emerged after the real stimulation and was less dependent on visual feedback. Instability was observed after the real stimulation in both EO (i.e., longer PL) and EC condition (i.e., increased ROM in the anterior–posterior axis). We confirm previous findings on cerebellar ataxia, showing that cerebellar balance instability does not improve with visual feedback compensation [33].

The observed wider base of support may be the expression of a stabilization strategy of the cerebellar group while walking, which is a predominant characteristic of gait in cerebellar ataxia (CA), [36]. Similarly, the observed increased variability in stride length and velocity matches the variable timing and spatial irregularity of foot placement in CA [36]. Increased gait variability seems to be the predominant emerging feature of the cerebellar inhibited group, as observed also in the kinematic of the knee (i.e., knee angle at foot elevation) and ankle angles (i.e., maximum and average angles). Contrary to expectations, these features emerged just in the EC trials. This may be the result of a suboptimal stimulation of the cerebellum, which is a deeper brain structure compared to the PPC, and thus more challenging to be reached with a figure-of-eight TMS coil [26]. The effects of stimulation may have become evident due to the summative effects of stimulation and task challenge (i.e., eyes closed condition).

### PPC and Cerebellum: Differences and Overlaps

After real stimulation, PPC and cerebellar group performances differed in several ways. First, the performance in the distance estimation task after the stimulation: the PPC group showed a significant overestimation of distances in longer trials (i.e., 20 m distances) in the EC condition compared to the cerebellar group. In the cerebellar group, the error rate between the EC and EO conditions was steady and near zero. This finding highlights the predominant role of PPC in using sensation to relate the body to target positions when walking. To explain the ability to estimate distances walked when visual information is not provided, the existence of a locomotor body schema has been previously hypothesized [41]. According to this theory, internalized knowledge of body segment lengths and positions, along with the perceived flexo-extensions of lower limb joints while walking, allow to estimate travelled path distances [42]. As the internalized model of the body originates from PPC multisensory integration [43], the TMS inhibitory effect may have altered this capacity. Second, visual feedback plays a role in stability maintenance. PPC integrates visual feedback among the other sensory signals to keep balance. Thus, removing the compensatory role of vision in a balance task may disclose a PPC deficit. On the contrary, cerebellar balance deficits do not improve with visual feedback. Third, even if spatiotemporal and kinematic analogies emerged after cerebellar and PPC functional inhibition (see Fig. 7), two different tendencies can be observed: PPC group was mainly affected in the temporal features of the eyes closed walking (i.e., cadence; speed; stance time; step time) while the cerebellar group showed a greater impact on spatial ones (i.e., stride length and step width). While in PPC increased time variability may result from disturbances of sensory feedback integrations [44], cerebellar wide-based walking and variable step length may be indices of the need for stability during locomotion.

## Limitations and Future Directions

This study has a few limitations to point out. First, we couldn’t use a system of neuro-navigation to target the sites of stimulation. These data should be replicated using neuro-navigation to spot with higher consistency the sites of stimulation. Second, the coil we adopted (i.e., figure-of-eight coil) is not the recommended one to reach the cerebellum: a double cone coil would be better to reach this and other deeper brain structures. However, some authors reported that double cone coil stimulation often led to pain and discomfort in neck muscles [26]. Additionally, many studies succeeded in stimulating the cerebellum by adopting a figure of eight coil [45]. Future studies should address the problem of tasks' order presentation, to ensure the absence of related biases, by randomizing the order of tasks between participants and increase the sample size. Lastly, we did not assess potential effects of the stimulation on the upper limb. The same paradigm might in future studies assess variations in upper limb kinematics following inhibitory rTMS of cerebellum and PPC.

## Conclusions

This study provides distinguishing motor and motor-related cognitive parameters following PPC and cerebellar functional inhibition. Visual feedback role in balance control, proprioceptive-guided distance estimation, and the prevalence of gait variability in spatial *vs* temporal parameters may be valuable indices to disentangle cerebellar *vs* parietal sensorimotor integration deficit. Clinical practice can benefit from these results: i) new assessment procedures could be developed considering the disclosing diagnostic potential of gait and stability parameters; ii) the compensatory role of visual feedback can mask eventual PPC-related motor deficits, thus, differences between EO and EC performances should be tested; iii) tasks as the distance estimation by walking are easy to administer, reliable and can be engaging for children. This type of paradigm looking for distinguishing similar clinical conditions can be used to improve differential diagnosis and enhance tailored rehabilitation.

## Supplementary Information

Below is the link to the electronic supplementary material.

Supplementary file1 (DOC 220 KB)

Supplementary file2 (XLSX 10 KB) S1 Table. Balance parameters: mean values

Supplementary file3 (XLSX 14 KB) S2 Table. Spatiotemporal parameters: mean values 

Supplementary file4 (XLSX 18 KB) S3 Table. Kinematic parameters: mean values

Supplementary file5 (XLSX 10 KB) S4 Table. Distance estimation task: mean and median delta values

## Data Availability

Data is provided within the manuscript or supplementary information files. Additional data that support the findings of this study are available on request from the corresponding author. The data are not publicly available due to privacy or ethical restrictions.
